# Amino acid-based surfactants: sustainable synthesis and antimicrobial mechanisms

**DOI:** 10.3762/bjoc.22.85

**Published:** 2026-07-16

**Authors:** Rafaela Gomes Bezerra, Lourdes Pérez, Francisco Fábio Oliveira de Sousa

**Affiliations:** 1 Graduate Program on Pharmaceutical Innovation, Department of Biological & Health Sciences, Federal University of Amapá (UNIFAP), Amapá, Macapá, Brazil; 2 Department of Pharmacy (DFAR), Faculty of Pharmacy, Dentistry and Nursing (FFOE), Federal University of Ceará (UFC), Fortaleza, Ceará, Brazil; 3 Department of Surfactants and Nanobiotechnology, Instituto de Química Avanzada de Cataluña, Centro Superior de Investigaciones Científicas (IQAC-CSIC), Barcelona, Spain

**Keywords:** amino acids, antibiofilm, antimicrobial, biodegradable, surfactants

## Abstract

Antimicrobial therapy is becoming increasingly ineffective over infections due to the gradual microbial resistance to conventional treatments. This aspect highlights the urgent need for the development of new antimicrobial agents. In this context, the amino acid-derived surfactants have proven to be a promising alternative to eradicate numerous microorganisms and their biofilms. The antimicrobial action of these agents can be explained by their amphiphilic structure, a configuration that allows interactions with structural components, such as the microorganism membranes and extracellular matrices of biofilms, promoting destructive effects on the cellular integrity and microbial vital processes. In addition, these surfactants can be easily synthesized using green chemistry principles, are biodegradable and more biocompatible than commercial quaternary ammonium surfactants. This paper aimed to elucidate the relevance of amino acid-derived surfactants with antimicrobial properties, presenting their structural compositions and connecting to the novel evidences on their mechanism of action. An integrative review was conducted, drawing upon findings from scientific articles published in the last 20 years (between 2005 and 2025), exclusively in English, retrieved from the PubMed database. This research employs a qualitative approach, adopting a basic research design with descriptive objectives, using a bibliographic method to explore the topic. The results indicate that the search for new amino acid-derived surfactants with antimicrobial activity has gradually grown over the last years. These molecules have shown promising characteristics, singular structures leading to innovative mechanisms of action, capable of overcoming the defenses of resistant microorganisms. In conclusion, the ability of these compounds to inhibit the growth of bacteria, yeasts, and fungi has been demonstrated, offering an effective approach to prevent and combat the spread of infections, especially in the context of microbial resistance.

## Introduction

Traditional antimicrobial therapies are becoming progressively less effective against bacterial and fungal infections, primarily due to the escalating prevalence of antimicrobial resistance among the pathogens, particularly in Gram-positive bacteria (e.g., methicillin-resistant *Staphylococcus aureus*, vancomycin-resistant *Enterococcus* sp.), Gram-negative bacteria (e.g., *Pseudomonas aeruginosa*, *Acinetobacter baumannii*) and fungi (e.g., *Candida auris*) [1] combined with the unreasonable use of antimicrobial therapies. Faced with this challenge, which brings serious risks to society, the development of new antimicrobial agents is urgently necessary [1–2].

Novel therapeutic strategies to combat resistant microorganisms are being extensively studied. Among those, the synthetic development of amino acid-derived surfactants is noteworthy [3]. These agents have shown to be a promising alternative to conventional antimicrobials, specifically those synthesized from lysine, arginine, and histidine, due to their direct membrane-disruptive activity, low propensity for resistance, and enhanced biodegradability compared to conventional surfactants like quaternary ammonium compounds [3–6].

**Figure 1:**
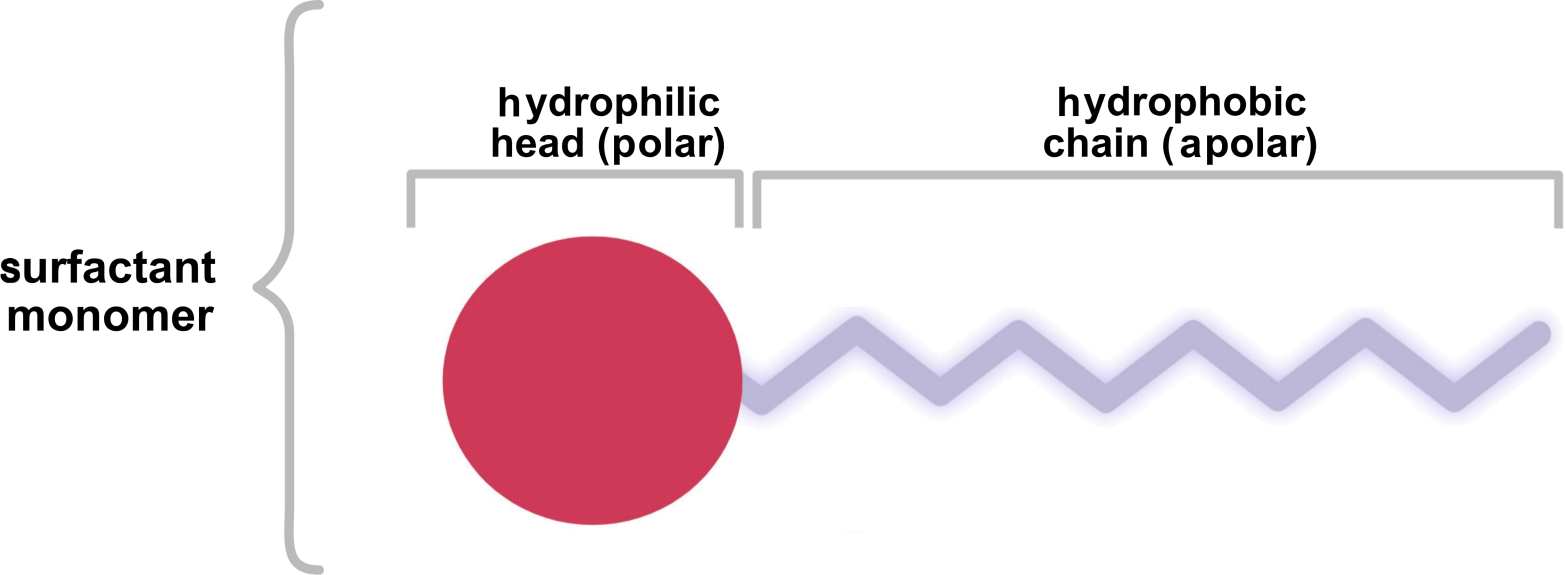
General structure of a surfactant monomer. The monomer consists of a hydrophilic (polar) head and a hydrophobic (apolar) chain. Figure 1 was created by using Canva; © Rafaela Gomes Bezerra via Canva.com. This content is not subject to CC BY 4.0.

Surfactants are commonly referred to by a range of synonyms, including surface-active agents, wetting agents, detergents, and emulsifiers. These terms underscore their unique ability to modulate surface tension and facilitate the interactions across diverse phase boundaries, making them versatile components in several applications [7]. These agents are classified as amphiphiles due to the presence of hydrophilic (polar) and hydrophobic (apolar) groups in their chemical structure (Figure 1). Moreover, they can be of biological origin (biosurfactants) or synthetic, from non-renewable or renewable sources, for instance made of amino acids [8–10].

**Figure 2:**
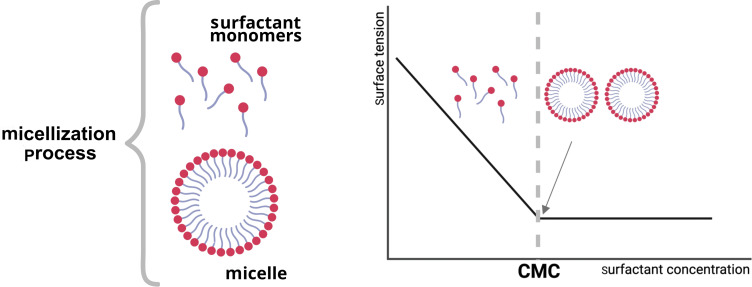
Micellization process. Surfactant monomers progressively reduce surface tension until reaching the critical micelle concentration (CMC), above which they self-assemble into micelles with hydrophobic tails oriented inward and hydrophilic heads exposed to the aqueous medium. Figure 2 was created by using Canva; © Rafaela Gomes Bezerra via Canva.com. This content is not subject to CC BY 4.0.

The tensoactive or surfactant is a compound distinguished by its capacity to modify the interfacial and surface property of a liquid. Their amphiphilic property is marked by a hydrophobic pole with a high affinity for organic solvents and a hydrophilic pole with a high affinity for water [11]. This aspect enables the surfactant monomers to orient themselves at the interface between two immiscible phases, such as oil and water. This positioning facilitates the reduction of surface tension and promotes the micellization process [12]. The formation of micelles starts when the surfactants reach the critical micellar concentration (CMC, Figure 2) [12–13].

These compounds can be classified according to the charge of the polar terminal groups in: anionic, cationic, nonionic, and zwitterionic [8]. Cationic surfactants are among the most used surfactants, both synthetic and from renewable sources. Despite their extraordinary antimicrobial properties, they are the most irritating category for skin and mucous membranes, which limits their application in food and medicinal products [12,14–15].

Cationic amino acid-derived surfactants, obtained from renewable sources, have gained great attention due to their superior biodegradability [6], good antimicrobial activity [3], and lower cytotoxicity [16–17]. These surfactants are derived from different amino acids, especially basic amino acids such as lysine and arginine, which can be combined in diverse structural arrangements, together with fatty acids or other lipophilic chains [18–19]. Upon the introduction of a reactive molecule with a hydrophobic chain, such as fatty acids, esters, amines and alcohols, through alkyl ester or amide bonds, amino acids having at least two functional groups (carboxylic and amino groups), can be easily converted into single-chain surfactants [3,5]. In addition to the simplified synthesis procedures, these compounds have some of the fundamental requirements for clinical application such as low toxicity, biocompatibility and high biodegradability [6,17,20].

Recent studies highlighted that the antimicrobial efficacy of these surfactants is highly dependent on structural features, including hydrophobic tail length (C12–C16 chains show optimal activity) [18–19]; cationic charge localization (e.g., arginine’s guanide group vs lysine’s primary amine) [21–22] and self-assembly properties (e.g., CMC) that influence biofilm penetration [12–13].

For instance, arginine-derived surfactants exhibit superior activity against ESKAPE pathogens due to their stronger electrostatic interactions with anionic phospholipids in bacterial membranes [23]. In contrast, lysine-based surfactants demonstrate broader-spectrum effects, including antibiofilm and antifungal properties [24]. Notably, these compounds also disrupt microbial membranes via lytic or non-lytic mechanisms (e.g., pore formation, lipid extraction, or inhibition of essential enzymes like ATP synthases) [25–28].

The antimicrobial activity can be explained by their cationic charge and amphipathic structure [21–22]. This configuration allows these compounds to give rise to electrostatic and hydrophobic interactions with the cell membranes of microorganisms, which are mainly formed by a lipid bilayer [23]. The studies on the antimicrobial properties of surfactants are promising, offering novel possibilities for anti-infective agents [24]. Surfactants act mainly by the destabilization of the cell membrane, which compromises the selectivity and homeostasis of the microorganism; they also act with the inhibition of essential enzymatic functions by specific interactions with proteins or nucleic acids among other mechanisms that will be described in the following sections [25–27].

Accordingly, this review aims to provide an overview of the relevance of these molecules and their suitability to combat bacterial and fungal infections, especially in face of the increased prevalence of resistant pathogens [29]. The amino acid-derived surfactants have demonstrated to be effective over a wide range of microbial strains and are found remarkably more biocompatible and biodegradable than synthetic compounds, being therefore more environmentally friendly [17,30].

The general objective of the research was to understand, according to the amino acid used for synthesis, the potential of the surfactants obtained in terms of its spectrum of action against strains of bacteria, yeasts or fungi [31]. The starting question was: what are the most used amino acids for the synthesis of surfactants and what are their functional characteristics, effect and spectrum over potentially resistant bacteria, yeasts and fungi?

To this end, the following specific objectives have been defined: a) To understand the molecular properties of amino acid-derived surfactants related to their antimicrobial activity; b) To describe their possible mechanisms of action in the interaction with microorganisms; and c) To present the novel approaches and perspectives on the use of these innovative molecules.

## Materials and Methods

The methodology for this integrative review was developed in accordance with the PRISMA (Preferred Reporting Items for Systematic Reviews and Meta-Analyses) guidelines [32]. The research adopted a qualitative framework to refine the investigation problem. The objectives are descriptive, as all findings were thoroughly documented with proper authorship citations [31–32].

**Figure 3:**
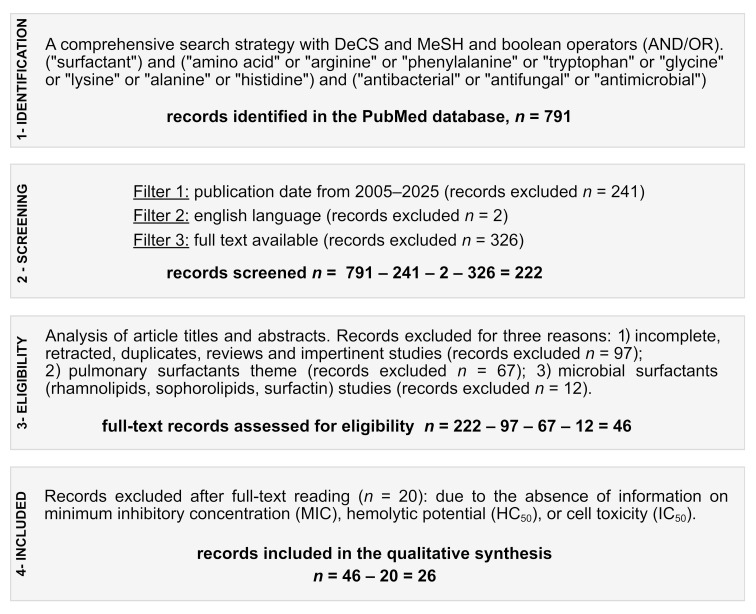
Study selection flowchart based on PRISMA guidelines. The process includes identification, screening, eligibility, and inclusion phases. Records were filtered by publication date (2005–2025), language (English), and availability of full text. Articles were excluded based on irrelevance, duplication, or being reviews, patents or books. Final selection included 26 studies with in vitro and/or in vivo evaluation of antimicrobial and/or cytotoxic activity. Figure 3 was created by using Canva; © Rafaela Gomes Bezerra via Canva.com. This content is not subject to CC BY 4.0.

The process was divided into four main phases: identification, screening, eligibility and inclusion as illustrated in the flowchart (Figure 3) [32]. In the identification phase, a comprehensive search strategy was conducted using specific health science descriptors (DeCS) combined with Boolean operators (AND/OR). This search was conducted in the PubMed database, resulting in an initial of 791 records [32–33].

The screening phase involved applying three filters to refine the results. The first filter excluded articles published outside the timeframe of 2005 to 2025, leading to the removal of 241 records. The second filter limited the selection to articles written in English, excluding two additional records. The third filter prioritized articles with full-text access, resulting in the exclusion of 313 records. After applying these filters, 222 records remained for further evaluation [32].

In the eligibility phase, titles and abstracts of the remaining articles were analyzed. Studies were excluded based on three reasons: (1) Incomplete, retracted, duplicates, reviews and impertinent studies (*n* = 97) ; (2) Pulmonary surfactants studies (*n* = 67) and (3) Microbial surfactants (rhamnolipids, sophorolipids, surfactin) studies (*n* = 12). Records excluded *n* = 176. This step reduced the selection to 46 eligible articles [32–33].

Finally, during the inclusion phase, full-text analysis was conducted on the selected articles. Records excluded *n* = 20 for the absence of information on minimum inhibitory concentration (MIC) or hemolytic potential (HC_50_: refers to the concentration of a substance that causes 50% hemolysis of red blood cells) or toxicity in cells (IC_50_: refers to the concentration of a substance that inhibits a biological or biochemical function by 50%). After this selection process, 26 studies were included in the integrative review [32,34].

The studies that met the inclusion criteria were evaluated and classified according to their methodological rigor, considering the characteristics of each study, using integrative review assessment tools [31–32,35].

## Review

The outcomes presented by the 26 selected scientific studies were of great relevance to meet the intended objectives. In general, these results contribute to evidence and validate that amino acid-derived surfactants can interact and stabilize interfaces and be functionally active between different phases, enabling them to have directly a great antimicrobial activity at the membrane level [19,21]. The effect is closely related to the structural characteristics and amphiphilic molecular properties dependent on the type of amino acid and lipid chain used in the synthesis of the surfactant [3,21].

Using the search descriptors afore mentioned, it was possible to find 222 studies within the PubMed database (an average of 11 manuscripts per year). In addition, the highest number of publications was observed in 2015, 2018 and 2021. However, regarding this review, only 26 were selected, such as shown in the flowchart of Figure 3.

### General structure and molecular properties of amino acid-derived surfactants

**Figure 4:**
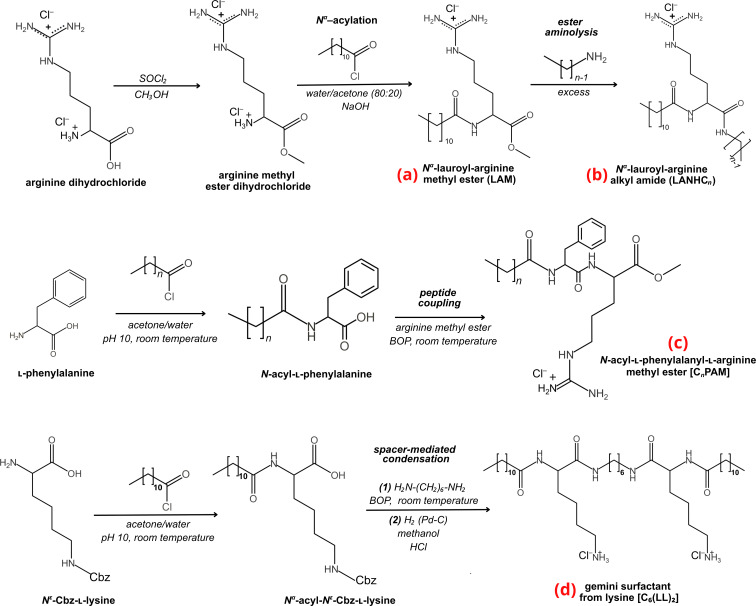
Representative synthetic routes illustrating the main structural classes of amino acid-based surfactants. (a) Single-chain derivatives prepared by N-acylation of amino acid esters using fatty acid chlorides under basic conditions; (b) double-chain surfactants obtained by ester aminolysis (secondary alkylation) of *N*^α^-lauroylarginine methyl ester (LAM) with long-chain amines, yielding *N*^α^-lauroylarginine alkyl amides (LANHC*_n_*); (c) diamino acid surfactants synthesized by peptide coupling between *N*-acylamino acids and amino acid esters using carbodiimide-based activation and (d) gemini surfactants derived from lysine through spacer-mediated condensation with α,ω-diamines, followed by deprotection. Figure 4 was created by using Canva; © Rafaela Gomes Bezerra via Canva.com. This content is not subject to CC BY 4.0.

Several molecular structures of the cationic amino acid-based surfactants are reported in the literature (Figure 4): (a) single chain surfactants with a single amino acid on the polar head and one alkyl chain, (b) double chain surfactants consisting of two alkyl chains linked to a single amino acid, (c) single alkyl chain surfactants with two amino acids on the polar head and (d) gemini surfactants containing two polar heads and two alkyl chains. This allows determination of the effects of different molecular structures (charge density, alkyl chain length/position and presence of aromatic groups) on the antimicrobial efficiency of these amphiphilic small molecules (Table 1) [4].

These structural classes are typically obtained through complementary synthetic strategies involving N-acylation and N-alkylation reactions. N-Alkylation is mainly achieved either by reductive amination with long-chain aldehydes followed by chemical reduction, or by direct nucleophilic substitution using activated alkyl halides or sulfonates [6,10,13]. Reductive amination generally affords improved control over mono-N-alkylation and moderate to high yields, whereas direct alkylation requires strict stoichiometric and reaction control to minimize overalkylation and side reactions [13]. In addition, double-chain derivatives (e.g., LANHC*_n_*) are commonly obtained via aminolysis of methyl esters with long-chain amines under thermal conditions [36].

Carboxylic acid activation and amide bond formation are commonly performed using acyl chlorides in Schotten–Baumann-type reactions or carbodiimide-mediated coupling agents such as DCC or EDC, providing high conversions under relatively mild conditions [3,6]. Reported yields generally range from moderate to high values depending on amino acid functionality, alkyl chain length, and purification strategy [10,37].

For structurally more complex surfactants, especially those derived from multifunctional amino acids such as lysine, arginine, and histidine, selective functionalization often requires the use of protecting groups, including Boc (*tert*-butyloxycarbonyl), Fmoc (fluorenylmethyloxycarbonyl), Cbz (benzyloxycarbonyl), and ester-based strategies, to ensure regioselectivity and controlled coupling [3,13]. Although effective, these approaches increase synthetic complexity and may limit large-scale applicability.

Purification is commonly achieved by acid–base extraction, selective precipitation, salt formation, and crystallization, while chromatographic techniques are mainly restricted to laboratory-scale studies [38]. Scalable processes preferentially rely on phase separation and crystallization to improve economic and environmental viability.

The diversity of these molecular architectures enables systematic evaluation of the effects of charge density, alkyl chain length and position, and aromatic moieties on antimicrobial performance, as extensively documented by Pérez and co-workers [4,12]. From a translational perspective, *N*-acylamino acid surfactants such as glycinates, sarcosinates, alaninates, and glutamates currently exhibit the highest potential for clinical and industrial applications, owing to their favorable safety profiles, biodegradability, and established large-scale production, while arginine- and lysine-based cationic derivatives remain promising for advanced biomedical uses.

Additionally, small variations in the structure of the surfactant, can be a strategy to improve the use of these compounds [39]. This aspect has been demonstrated in studies of compounds such as diacyl-glycerol-arginine (1414R,1212: arginine-based surfactants bearing two C14 and two C12 alkyl chains, respectively), where the substitution of the *N*^α^-amine for a *N*^α^-acetylamide group, drastically changed its antimicrobial activity against *Acinetobacter baumannii, S. aureus, Bacillus cereus* and *Brochothrix thermosphacta* [39–40].

#### Single-chain monoamino acid surfactants

Single-chain surfactants are the simplest chemical structure (Figure 4a). The simple structure offers significant economic and environmental advantages, making them highly desirable compounds. Their synthesis aligns with current environmental regulations by using renewable starting materials and reducing significantly the need for hazardous solvents, making them a very sustainable option [3]. For instance, tryptophan and phenylalanine-based surfactants have been prepared using a green method that employs renewable starting materials and involves a streamlined two-step synthesis that avoids the use of heavy organic solvents and protected amino acid groups, enhancing its environmental sustainability [5–6].

Life cycle analyses confirm their advantages over petrochemical-derived surfactants. Pinazo, Manresa et al. (2016) highlight that these surfactants can be synthesized using green chemistry principles, including the use of renewable starting materials, high atom economy (85–92%), and elimination of hazardous by-products, while maintaining effective antimicrobial activity [3]. Comparative studies show that these surfactants maintain effective antimicrobial activity, with MIC values typically ranging from 2 to 64 μg/mL against *Staphylococcus aureus*, depending on the specific surfactant structure [6].

#### Double-chain surfactants

Double-chain surfactants (Figure 4b) are characterized by the presence of two hydrophobic chains linked to a single polar head group, resulting in enhanced amphiphilicity, lower critical micelle concentrations (CMC), and improved membrane-interaction properties when compared with their single-chain counterparts [16,41]. A classic example is the quaternary ammonium compound didodecyldimethylammonium bromide (DDAB), one of the most widely studied double-chain cationic surfactants due to its strong antimicrobial and biofilm-control activity [42–43]. However, DDAB lacks the biodegradability and biocompatibility typically associated with amino acid-derived surfactants and, similarly to other conventional quaternary ammonium compounds, may present higher environmental persistence and toxicity [17,29].

In contrast, amino acid-based double-chain surfactants containing residues such as arginine or lysine combine the antimicrobial efficacy of conventional cationic surfactants with improved environmental and toxicological profiles. Recent studies have demonstrated that these compounds exhibit lower cytotoxicity, enhanced biodegradability, and significantly reduced aquatic toxicity compared with conventional quaternary ammonium compounds [16–17,29].

The synthesis of amino acid-based double-chain surfactants generally involves the covalent attachment of hydrophobic chains to amino acid-derived head groups through amide, ester, or alkyl linkages while preserving the structural contribution of the natural amino acid moiety [44–45].

For example, arginine-based double-chain surfactants of the LANHC*_n_* series are commonly prepared by aminolysis of *N*^α^-lauroylarginine methyl ester (LAM) with long-chain amines, generating asymmetric double-chain structures with tunable physicochemical and biological properties [44–45].

This dual-chain design imparts significantly higher hydrophobicity compared with single-chain analogues, lowering the critical micelle concentration (CMC) and improving interfacial stability properties critical for the formation of stable micelles and bilayers [46]. This effect is illustrated by the LANHC series (Table 1, entries 3–6), which exhibits CMC values between 0.032 and 0.16 mM [44], substantially lower than those observed for the single-chain surfactants LAM and LAE (Table 1, entries 1 and 2), whose CMC values range from 5.3–6.1 mM and 5.8–6.5 mM, respectively [47–51].

**Table 1:** Physicochemical properties and antimicrobial activity of amino acid-based surfactants.

Entry	AA*	Type	Acronym	CMC (mM)	MIC (μg/mL)	Representative microorganisms

1	Arg	single-chain (lauroyl)	LAM [48,50]	5.3–6.1	2–32	*S. aureus, E. coli*
2	Arg	single-chain (lauroyl)	LAE [47,51]	5.8–6.5	8–64	*L. monocytogenes,* *S. typhimurium,* *Y. enterocolitica*
3	Arg	double-chain	LANHC10 [44]	0.13–0.16	4–32	*S. aureus, E. coli*
4	Arg	double-chain	LANHC12 [44]	0.049–0.061	8–64	*S. aureus, E. coli*
5	Arg	double-chain	LANHC14 [44]	0.032–0.038	16–128	*E. coli*
6	Arg	double-chain	LANHC18 [44]	0.081–0.099	64–256	*E. coli*
7	Arg	double-chain glycerolipidic	88R [55]	0.07	4–64	Gram+ bacteria
8	Arg	double-chain glycerolipidic	1010R [39]	0.006	16–256	Gram+ bacteria
9	Arg	double-chain glycerolipidic	1212RAc [39]	0.008	32–256	Gram+ bacteria
10	Arg	double-chain glycerolipidic	1414RAc [39]	0.002–0.008	8–256	Gram+ bacteria
11	Arg	gemini	C3(OA)2 [16]	8.0	32–128	*S. aureus*
12	Arg	gemini	C3(CA)2 [16]	0.12–0.18	8–64	*S. aureus, E. coli*
13	Arg	gemini	C3(LA)2 [16]	0.018–0.030	8–64	*S. aureus, E. coli*
14	Arg	gemini	C6(LA)2 [16]	0.010–0.025	16–128	*E. coli*
15	Arg	gemini	C9(LA)2 [16]	0.006–0.015	4–64	*E. coli*
16	Arg	benzoyl-arginine	Bz-Arg-NHC10 [56]	0.14	27.6–95	*S. aureus*
17	Lys	single-chain	LLM [57]	7.2	11–94	*S. aureus, E. coli*
18	Lys	single-chain	LKM [57]	5.5–8.5	5–47	*S. aureus*
19	Lys	single-chain	TMKM [57]	3.0	6–50	Gram+ bacteria
20	His	gemini	DMHysNHC14 [30]	5.2	4–70	*S. aureus*
21	Leu	benzyl leucine	C10–C14 LeuBENZ [58]	0.04–2.04	1–128	*S. aureus, E. coli*
22	Phe	aromatic	Cn-Phe [59]	0.011–3.9	6–256	*S. aureus, E. coli*
23	Trp	aromatic	Cn-Trp [60]	0.42–6	1.8–185	*S. aureus*
24	Tyr	aromatic	Cn-Tyr [61]	0.013–1.94	2–1500	Gram+ bacteria

*AA, amino acid; Arg, arginine; Lys, lysine; His, histidine; Leu, leucine; Phe, phenylalanine; Trp, tryptophan; Tyr, tyrosine; CMC, critical micelle concentration; MIC, minimum inhibitory concentration; Gram+, Gram-positive bacteria. Data are presented as ranges or single values according to the original references.

These surfactants excel as broad-spectrum disinfectants, upon their strong membrane-disruptive action against pathogens [3,23]. Additionally, their ability to stabilize nanoparticles and facilitate transfection in molecular biology (e.g., gene delivery) stems from their amphiphilic balance, which enables efficient interaction with both hydrophobic surfaces and charged biomolecules [23,52]. By bridging synthetic and bio-inspired design, double-chain amino acid surfactants offer tailored solutions for applications demanding both potency and biocompatibility [5,53].

These structurally advanced surfactants bridge the gap between conventional quaternary ammonium compounds and bio-based antimicrobial agents. Amino acid-derived double-chain surfactants display strong antimicrobial activity while maintaining improved environmental compatibility. For example, the LANHC series (Table 1, entries 3–6) exhibits MIC values ranging from 4 to 256 μg/mL depending on alkyl chain length and target microorganism, together with markedly lower CMC values (0.032–0.16 mM) than those observed for single-chain arginine surfactants such as LAM and LAE (Table 1, entries 1 and 2; CMC 5.3–6.5 mM). Similarly, arginine-based gemini surfactants (Table 1, entries 12–15) combine low CMC values (0.006–0.18 mM) with MIC values of 4–128 μg/mL against *S. aureus* and *E. coli*. These physicochemical characteristics favor membrane interaction, self-assembly, and the formation of stable supramolecular systems, supporting their potential for pharmaceutical and biomedical applications [23,54].

Among double-chain arginine surfactants, the LANHC series (Table 1, entries 3–6) demonstrates a clear chain-length dependence on antimicrobial activity. The shortest homologue, LANHC10 (Table 1, entry 3), exhibits MIC values of 4–32 μg/mL against both *S. aureus* and *E. coli*, while longer chains (LANHC14, Table 1, entry 5) show reduced activity against Gram-negative bacteria (MIC 16–128 μg/mL) and LANHC18 (Table 1, entry 6) is only active against *E. coli* at higher concentrations (64–256 μg/mL) [44]. This trend correlates with increasing hydrophobicity and lower CMC values, which reduce monomer bioavailability.

Glycerolipidic arginine derivatives (Table 1, entries 7–10) also display potent activity against Gram-positive bacteria. The 88R homologue (Table 1, entry 7) shows the lowest MIC values (4–64 μg/mL) [55], whereas 1414RAc (Table 1, entry 10) exhibits a broader MIC range (8–256 μg/mL) but with substantially lower CMC values (0.002–0.008 mM), reflecting its higher hydrophobicity [39]. These compounds illustrate how the introduction of two alkyl chains and a glycerol backbone modulates both aggregation behavior and biological activity.

The strategic incorporation of histidine creates pH-responsive systems, with protonation studies showing complete antimicrobial activation below pH 6.5 [62]. Molecular dynamics simulations have demonstrated that phenylalanine-modified variants exhibit enhanced penetration into lipid bilayers, which has been attributed to the favorable partitioning of the aromatic side chain within the hydrophobic core of the membrane [63–64]. This property correlates with their improved efficacy against *P. aeruginosa* [16,59].

This adaptability allows tailored designs for specific interactions, balancing charge and hydrophobicity. Synthesis typically involves stepwise coupling strategies using protecting groups (e.g., Fmoc or Boc) to ensure precise amino acid incorporation, followed by amidation or esterification reactions to anchor the alkyl chain [65]. Such controlled synthetic pathways enable the creation of structurally refined surfactants, optimized for applications ranging from antimicrobial agents to biomolecular delivery systems [66].

#### Single-chain diamino acid surfactants

Single-chain surfactants incorporating two amino acids within their polar head (Figure 4c) are a versatile class of amphiphiles, structurally defined by a single hydrophobic alkyl chain connected to a polar region housing dual amino acid residues [67]. These molecules share similarities with short lipoamino acids, where the integration of two amino acids amplifies the charge density and their functionality [13].

Examples include surfactants with homotypic pairs (e.g., dual lysine residues) or heterotypic combinations (e.g., lysine paired with histidine), while structural diversity can be further expanded by incorporating aromatic amino acids like phenylalanine to modulate the hydrophobicity [62,68–69]. A key advantage lies in their enhanced cationic charge density compared to single-amino-acid surfactants, which strengthens electrostatic interactions with negatively charged bacterial membranes particularly effective against Gram-negative pathogens [3,65].

Structural analyses reveal these surfactants achieve 1.5–2 times greater charge density than their mono-amino acid counterparts [13]. This is exemplified by dilysine surfactants (Table 1, entries 17–19) demonstrating low MIC values (5–50 μg/mL) against representative bacterial strains [57].

Among lysine-based single-chain surfactants, the trimethylated derivative TMKM (Table 1, entry 19) deserves special attention. Unlike its non-methylated counterparts LLM and LKM (Table 1, entries 17 and 18), TMKM bears a fixed positive charge on the α-amino group, independent of pH. This structural modification results in consistent activity against Gram-positive bacteria (MIC 6–50 μg/mL) without requiring protonation at physiological pH, making it particularly attractive for formulations where pH stability is critical [57].

#### Gemini surfactants

Gemini surfactants (Figure 4d), a sophisticated class of amphiphiles, are defined by their unique structure: two hydrophobic chains covalently bonded to a shared nitrogen atom within the polar headgroup, which is often amino acid derived [70–71]. These dual chains are interconnected by a rigid or flexible spacer (e.g., ethylene glycol), enabling precise control over molecular geometry and aggregation behavior. A key example includes lysine-based gemini surfactants, where the amino acid polar heads are linked via spacers to optimize the charge distribution and bioactivity [57,72].

Representing the most sophisticated architecture, these surfactants exhibit exceptional performance metrics: CMC values 100–1000 times lower than monomeric surfactants; thermal stability up to 300 °C [70] and MIC values reported as low as 4 μg/mL against MRSA depending on the molecular structure, with C3(CA)2 (Table 1, entry 12) and C3(LA)2 (Table 1, entry 13) showing potent activity (MIC 8–64 μg/mL) against both *S. aureus* and *E. coli* (Table 1, entries 11–15) [16,72–74].

Within the arginine-based gemini series (Table 1, entries 11–15), chain length and spacer geometry play critical roles. The C3(OA)2 homologue (Table 1, entry 11), with octyl chains, shows moderate activity (MIC 32–128 μg/mL against *S. aureus*) and a relatively high CMC (8.0 mM). In contrast, C3(CA)2 (Table 1, entry 12) and C3(LA)2 (Table 1, entry 13), with lauroyl chains, exhibit substantially lower MIC values (8–64 μg/mL) against both *S. aureus* and *E. coli*, together with 40- to 400-fold lower CMC values [16].

The C6(LA)2 homologue (Table 1, entry 14), with a longer spacer, shows slightly higher MIC values (16–128 μg/mL) against *E. coli*, while C9(LA)2 (Table 1, entry 15) is the most potent among the series, with MIC values as low as 4–64 μg/mL against *E*. *coli* and a CMC in the micromolar range (0.006–0.015 mM) [16].

Beyond the most studied amino acids, leucine-based surfactants also exhibit structure-dependent antimicrobial activity. The benzyl leucine series C10–C14 LeuBENZ (Table 1, entry 21) shows MIC values ranging from 1 to 128 μg/mL against *S. aureus* and *E. coli,* with a strong dependence on alkyl chain length. The CMC varies considerably across this series (0.04–2.04 mM), reflecting the influence of the hydrophobic tail on both aggregation behavior and biological activity [58].

The synthesis of these molecules typically involves a two-step coupling process, often using geminal dihalides and functionalized amino acids. Nonetheless, some challenges such as by-product formation and high purification costs limit their scalability [75]. Despite these hurdles, their unmatched efficiency and versatility have driven their assimilation in pharmaceuticals (e.g., antimicrobials, gene delivery) and cosmetics, where their ability to destabilize microbial membranes and stabilize formulations aligns with the demands for high-performance, resistance-busting agents [71,76].

In summary, amino acid-derived surfactants represent a sustainable, adaptable class of amphiphiles, bridging bioinspiration and industrial utility [3,24,45,77]. Their structural diversity allows precise tuning for applications ranging from healthcare to environmental science, positioning them as the next-generation alternatives to conventional surfactants [3,24].

#### Amino acids and lipidic chains used in the composition of surfactants

Most surfactants available on the market are obtained from petroleum-derived compounds [8]. However, the growth of environmental awareness of consumers, combined with the implementation of stricter legislation aimed at environmental preservation, has driven the search for sustainable alternatives. As a result, amino acids surfactants have gained great prominence as viable substitutes for conventional products derived from non-renewable sources [3,5].

The diverse array of amino acids, varying in their polar, non-polar, acidic, or basic characteristics, enables to design a broad spectrum of cationic amphiphilic molecules with tailored specifications [3,78]. The antimicrobial performance of amino acid-based surfactants is governed by precise structural optimization of both polar headgroups and hydrophobic tails, as demonstrated by extensive structure–activity relationship studies.

The selection of amino acid moieties critically influences electrostatic interactions with microbial membranes, with arginine-derived surfactants exhibiting particularly strong antimicrobial activity due to the guanidinium group's enhanced cationic character. Quantitative studies reveal that arginine-containing surfactants typically display MIC values between 2 and 64 μg/mL depending on molecular architecture (Table 1, entries 1, 3, 7, 12, and 13) [47–48,50–51]. This enhanced activity stems from the guanidinium group's +1.5 charge density and unique bidentate hydrogen bonding capability, which promotes stronger interactions with phosphate groups in microbial membranes.

Aromatic amino acids contribute significantly to membrane penetration through distinct mechanisms [28]. Surfactants incorporating phenylalanine (Table 1, entry 22) demonstrate significantly deeper insertion into lipid bilayers, as measured by fluorescence quenching studies, an effect attributed to the aromatic side chain [52], while tryptophan derivatives (Table 1, entry 23) show exceptional activity against MRSA with MIC values of 4–8 μg/mL [50,54–55], tyrosine-based surfactants (Table 1, entry 24) are primarily active against Gram-positive bacteria, with MIC values ranging from 2 to 1500 μg/mL depending on chain length [61]. These effects arise from combined hydrophobic interactions and π–π stacking with membrane components, with molecular dynamics simulations confirming 2–3-fold increases in membrane residence time for aromatic variants compared to aliphatic analogs as demonstrated by molecular dynamics simulations, the non-polar side chain of phenylalanine partitions favorably across the entire lipid bilayer, including its hydrophobic core [63–64].

The optimal hydrophobic chain length follows a well-defined parabolic relationship [47–48,51,56], with C12–C14 alkyl chains providing the ideal balance of membrane affinity and solubility. Systematic evaluations reveal that C12 chains typically yield MICs of 8–32 μg/mL with favorable hemolytic profiles (HC_50_ 200–300 μg/mL) [48,51], whereas C14 chains (log *P* ≈ 5.2) achieve greater potency (MIC 4–8 μg/mL) at the cost of increased cytotoxicity (HC_50_ 50–100 μg/mL) [47]. Shorter chains (C10) suffer from reduced membrane partitioning (≈30% lower incorporation by fluorescence anisotropy) [56], whereas longer chains (C16) predominantly form micelles, reducing bioavailable monomers as evidenced by CMC studies [47,56].

Comparative analyses demonstrate that arginine–C14 surfactants consistently outperform other configurations, showing four-fold greater potency than lysine derivatives and two-fold improved selectivity indices (HC_50_/MIC) versus phenylalanine analogs [57–58]. These structure–activity relationships are particularly evident against challenging pathogens, with arginine–C14 surfactants maintaining MICs of ≈8 μg/mL against drug-resistant *P. aeruginosa* strains [58].

The quantitative data establish clear design principles for optimizing both antimicrobial efficacy and biocompatibility, providing a robust framework for developing next-generation surfactant antimicrobials [59]. These findings underscore the importance of balanced molecular design, where strategic combinations of specific amino acid headgroups with precisely tuned alkyl chains yield optimal biological performance across diverse microbial targets [60].

#### Antimicrobial mechanisms of the amino acid-derived surfactants

Microorganisms, over time, have developed adaptive resistance mechanisms, such as the production of enzymes that degrade antibiotics (such as β-lactamases), modifications to the target site of antimicrobials, alteration on the cell membrane permeability and activation of efflux pumps [79]. The rapid development of antibiotic resistance among bacterial species is significantly facilitated by mobile genetic elements (MGEs), such as plasmids and transposons. These elements play a crucial role in the global health crisis by enabling the horizontal transfer of resistance genes across bacterial populations [2]. Faced with this challenge, due to their ability to interact with cell surfaces and membranes, amino acid-derived surfactants emerge as promising and biodegradable antimicrobial candidates [3].

#### Membrane disruption: electrostatic and hydrophobic interactions

The primary mechanism of action involves electrostatic attraction between the surfactant’s polar head (e.g., arginine’s guanidinium group, p*K*_a_ ≈ 12.5) and anionic microbial surface components – teichoic acids in Gram-positive bacteria (e.g., *Staphylococcus aureus*) or lipopolysaccharides in Gram-negatives (e.g., *Escherichia coli*) [80]. This interaction is stabilized by hydrogen bonding, followed by insertion of the hydrophobic tail (typically C8–C14) into the lipid bilayer, causing membrane destabilization and cell lysis [81].

The polar head of surfactants binds to bacterial membranes initially through electrostatic attraction between the surfactant’s positively charged groups (e.g., arginine’s guanidine, which remains protonated at physiological pH due to its high p*K*_a_) and the negatively charged microbial surface components (e.g., phospholipids, teichoic acids in Gram-positive bacteria, or lipopolysaccharides in Gram-negative bacteria) [21,80].

This initial interaction is reinforced by hydrogen bonds, anchoring the surfactant to the membrane. In Gram-positive bacteria, the absence of a complex outer membrane allows direct access, while in Gram-negatives, the physical barrier of the outer membrane reduces efficacy, requiring highly soluble and charged surfactants, such as LAM (Table 1, entry 1), for penetration [81–82]. After binding, the surfactant’s hydrophobic tails insert into the lipid bilayer, destabilizing the membrane structure and causing cellular content leakage, leading to the consequent microbial death [81].

Enhanced solubility and persistent positive charges found in arginine-containing molecules, such as LAM, amplify the membrane damage, while the surfactants’ physical disruption mechanism makes microbial resistance less likely to occur. Overall, the antimicrobial performance relies on the surfactants’ molecular architecture, charge preservation at physiological conditions, and their compatibility with delivery-system strategies [82–83].

Individually or combined, other amino acids such as lysine [68,84], tryptophan, phenylalanine [4,59], glycine [85], serine [86], cysteine, histidine, leucine and valine [56,87] are also used to synthesize different surfactants. The antimicrobial activity of these amino acid-based surfactants against various bacteria, yeasts and fungi strains depends primarily on the amino acid in the polar head. In a study with the arginine-derived surfactant Bz-Arg-NHC10 (Table 1, entry 16), it has been suggested that the benzoyl group present in the polar head improves its antimicrobial activity against Gram-negative strains (MIC 27.6–95 μg/mL against S. aureus) compared to other arginine-based agents [56].

Beyond the examples listed in Table 1, sodium lauroyl sarcosinate (sarkosyl) has also been explored in oral-care formulations. In an in vitro study, a dentifrice containing stabilized chlorine dioxide, sodium lauroyl sarcosinate, and sodium fluoride improved pellicle cleaning and inhibited oral polymicrobial biofilm regrowth, supporting its relevance in dental antibiofilm applications [88]. Lauroyl methionine exhibits potent action against *Staphylococcus aureus* (MIC = 16 μg/mL) with low cytotoxicity (IC_50_ > 500 μg/mL), indicating its suitability for dermatological formulations [89]. Meanwhile, LAM (Table 1, entry 1) demonstrates high efficacy against Gram-negative bacteria such as *E. coli* (MIC: 8 μg/mL) [82].

#### Biofilm penetration and synergistic effects

Amino acid-based surfactants exhibit unique capabilities in penetrating biofilm matrices, thereby enhancing the susceptibility of pathogens to co-administered antimicrobial agents. For example, arginine-tryptophan surfactants (LTAM) demonstrate potent activity against fluconazole-resistant *Candida* spp. (MIC: 8–12 μg/mL) by disrupting mitochondrial function and inducing apoptosis, while also exhibiting synergistic effects with amphotericin B [19]. Similarly, glycine-based solutions (4.5%) have proven effective in eradicating *Streptococcus* spp. and *Candida* spp. from medical devices, highlighting their utility in clinical settings [85].

Based on the results presented by Fait et al. (2023), the antifungal effect of arginine-based surfactants can also be explained by the detergent action on the plasmatic membrane lipids that induces the elimination of mixed micelles and macrovesicles and/or by the resulting apoptosis caused by membrane destabilization induced, for instance, by Bz-Arg-NHC*_n_* surfactant (*N*^α^-benzoyl-ʟ-arginine-alkylamide) [90].

An study with arginine-phenylalanine or arginine-tryptophan-derived surfactants (LPAM and LTAM) has shown that the antimicrobial efficacy of these surfactants can be potentiated with another antimicrobial, e.g., amphotericin B, against different resistant *Candida* spp. These surfactants were very effective against fluconazole-resistant Candida strains with remarkably low MIC values of 8.12 μg/mL. Their mechanism of action involves disrupting cell membranes and its permeability together with mitochondrial dysfunction, leading to fungal cell apoptosis. The compounds presented excellent biocompatibility and also dispersed resistant fungal biofilms at low concentrations (81.2 μg/mL), working synergistically with amphotericin B [19].

#### Chain length-dependent activity and CMC relationships

A comparative analysis of the CMCs and minimum inhibitory concentrations (MICs) of cationic surfactant analogues derived from ʟ-phenylalanine (C1–C20) and ʟ-tyrosine (C8–C14) esters against various bacterial strains, including *Staphylococcus aureus, Staphylococcus epidermidis, Bacillus cereus, Escherichia coli, Pseudomonas aeruginosa, Salmonella typhimurium*, and *Klebsiella pneumoniae*, revealed that surfactants with longer alkyl chains exhibited lower CMC values together with higher MIC values [59].

For instance, phenylalanine C12 ester shows enhanced activity against *S. aureus* (MIC: 25 μg/mL) compared to its more hydrophobic counterparts [59]. Conversely, surfactants exhibiting greater hydrophobic character and lower CMC values often display higher MICs, as illustrated by selected compounds in Table 1 (entries 5, 6, and 14), which combine low CMC values with MICs ranging from 16 to 256 μg/mL [59,91]. Similar results were reported by Birnie et al. (2000) for alkyl betaine and alkyl dimethylamine oxide series [92] and by Hafidi et al. (2023) for octyl esters and decyl chloride derivatives of ʟ-phenylglycine [92–93]. This phenomenon is attributed to the reduced number of monomers found at these concentrations, requiring higher surfactant concentrations to achieve the desired bactericidal effect [81,91].

These findings suggest that the antimicrobial properties of these surfactants are primarily attributed to their monomeric form rather than their micellar structure [23,67]. This insight highlights the importance of surfactant design for the optimization of their antimicrobial efficacy, particularly in the context of alkyl chain lengths, as they impact directly in the microbial interactions [67].

Furthermore, any emerging resistance comes with substantial fitness costs, as resistant strains demonstrate 30–40% reduced growth rates under optimal conditions [94]. These properties, particularly their effectiveness against biofilms and low resistance potential, position amino acid-based surfactants as promising solutions to the growing antimicrobial resistance crisis, especially for challenging biofilm-associated infections where traditional therapies often fail [16].

The combination of immediate membrane disruption with secondary intracellular effects and biofilm penetration represents a significant advance in antimicrobial design, offering potential solutions to some of the most pressing challenges in infection control [95–96].

#### Multitarget mechanisms and resistance

Emerging research demonstrates that amino acid-based surfactants employ sophisticated multitarget mechanisms, with in silico studies revealing specific inhibitory concentrations and binding affinities [16,60]. Molecular docking simulations demonstrated that these compounds bind to enzymes critical for microbial survival, such as DNA gyrase (involved in bacterial replication) and dihydrofolate reductase (DHFR) (key to nucleotide synthesis), inhibiting their activity through steric hindrance and hydrogen bonding [60,97].

The arginine-derived surfactant lauroyl-arginine ethyl ester shows notable DNA gyrase inhibition at 32 μg/mL (IC_50_ = 18.7 μM), effectively halting bacterial replication [97]. Tryptophan-based analogs exhibit strong binding to dihydrofolate reductase (DHFR) with a dissociation constant (KD) of 2.3 μM, disrupting folate metabolism in MRSA at concentrations as low as 8 μg/mL [60]. Flow cytometric analysis quantifies these effects, showing 78.4% DNA fragmentation in MRSA populations after 2-hour exposure to 16 μg/mL lauroyl-arginine surfactant [60]. These quantitative findings demonstrate dose-dependent, multitarget antimicrobial activity while maintaining selectivity (therapeutic index > 8 for most pathogens), highlighting their potential as resistance-resistant antimicrobial agents.

The in silico insights not only explain the synergistic efficacy of surfactants against resistant strains but also guide rational structural modulation, for example, adjusting hydrophobic chains or functional groups, to optimize their affinity over multiple targets, combining membrane disruption with enzymatic inhibition [97–99]. Other mechanisms are under investigation and may also be validated. This multifaceted approach, validated by computational tools, positions amino acid-based surfactants as promising agents to overcome resistance mechanisms and broaden their application in the next-generation antimicrobial therapies [97,100–101].

The relative contribution of these mechanisms varies by surfactant class, with single-chain variants relying predominantly (80–90%) on membrane disruption, while gemini surfactants exhibit a more balanced multimodal activity profile combining membrane effects (40%), biofilm penetration (30%) and intracellular targeting (30%) [95–96]. This multifaceted approach creates a formidable barrier against resistance development, with longitudinal studies showing bacteria require 20–30 generations to develop modest (2–4 fold) resistance, significantly slower than the 5–10 generations needed for conventional antibiotics [102].

#### Cytotoxicity of amino acid-derived surfactants

The amino acid-based surfactants have demonstrated powerful antimicrobial activity. Nonetheless, due to their charge and similarities to the cellular phospholipids, their use is limited due to their irritative and cytotoxic potential. Regarding the cytotoxicity, it is important to emphasize the induction of hemolysis [103], a significant phenomenon both in research and in practical applications, especially in the context of biomedicine [103].

Human erythrocytes, which lack internal organelles, serve as an ideal model for studying the interactions between surfactants and cell membranes [104]. The presence of surfactants in the cell membrane causes changes in the molecular organization, resulting in an increase in the membrane permeability and, eventually, cell lysis [105–106].

The safety profile of amino acid-based surfactants is commonly evaluated using two key parameters: the hemolytic concentration (HC_50_), which represents the surfactant concentration causing 50% lysis of erythrocytes, and the therapeutic index (TI), calculated as the ratio HC_50_/MIC against target microorganisms [103]. These metrics are particularly important as these surfactants demonstrate membrane-disruptive antimicrobial activity that can also affect mammalian cells. In other words, a high therapeutic index means a safer usage [107].

#### Hemolytic activity and structural dependence

The HC_50_ reflects erythrocyte compatibility, with human red blood cells serving as an ideal model due to their lack of internal organelles [104], while the TI quantifies the window between antimicrobial efficacy and cytotoxicity. Arginine-derived surfactants like *N*-lauroyl arginine ethyl ester (LAE) show favorable safety profiles with HC_50_ values typically >200 μg/mL and TI values of 24–48 against common pathogens [106,108].

Hemolysis is a major limitation of cationic amino acid surfactants, particularly those with longer hydrophobic chains [68]. For example, arginine-based surfactants like LAE and LAM exhibit moderate hemolysis (TI = 24–48) [106], while fluorinated pyridinium surfactants show significantly lower hemolytic activity due to tail fluorination [109].

#### Therapeutic index (TI) comparison across surfactant classes

The cytotoxicity of amino acid-based surfactants seems to be driven by the same structural features that affect the antimicrobial activity. In general, for cationic amino acid surfactants the cytotoxicity increases as the hydrophobic content of the molecule increases. The safety and efficacy profiles of surfactants vary significantly across different structural classes, as evidenced by their therapeutic indices (TI) and biological impacts [54,68].

Single-chain cationic surfactants like LAE and LAM demonstrate moderate TI values of 24–48 [106], offering the practical advantages of being food-safe at concentrations ≤200 ppm and exhibiting good biodegradability [108]. In contrast, conventional anionic surfactants such as sodium dodecyl sulfate (SDS) show poor safety margins with TI values <1 [110], coupled with significant cytotoxicity and environmental persistence.

More advanced architectures like gemini surfactants (e.g., C12-C6-C12 Br) achieve superior TI values ranging from 50 to over 200 [105,110], combining high antimicrobial potency with reduced critical micelle concentrations [111]. Fluorinated surfactants, while exhibiting excellent TI values >100 [109], present environmental concerns due to their persistence in ecosystems [112].

Vyas et al. (2006) reported that the hemolytic activity of partially fluorinated pyridinium surfactants was lower compared to cationic lysine surfactants. They found that as the degree of fluorination and the length of the hydrophobic tail increased, the hemolysis decreased dramatically. This suggests that modifying the surfactants' structure can lead to reduced toxicity towards erythrocytes, making them potentially safer for biomedical applications [109].

This comparative analysis highlights the critical balance between antimicrobial efficacy, mammalian cell safety, and environmental impact that must be considered when selecting surfactants for specific applications, with amino acid-based cationic surfactants offering a favorable middle ground between performance and biocompatibility.

#### Selectivity

In addition to the biodegradability, another major advantage of these surfactants is their ability to interact with bacterial membranes without compromising human cells due to the balance between hydrophobic and hydrophilic properties, as well as the type of bond present in the molecule, such as amides or esters [78]. LAE, a commercial cationic agent with low toxicity, has attracted increasing interest due to its improved antimicrobial activity [49,113].

Previous studies have shown that LAE binds to the acidic phospholipids and depolarizes the bacterial membrane, resulting in bacterial death [50,114–116]. In addition, LAE promotes the growth of gut microbiota. As a result, LAE is not only a potential surfactant against bacterial infection, but also a low-risk food additive (Table 1) [51,115].

#### Genotoxicity

A study of the genotoxicity of sodium dodecyl sulfate, an anionic surfactant, and a cationic surfactant based on glutamic acid reveals significant differences in their effects on maize. Sodium dodecyl sulfate shows considerable toxicity, leading to reduced root growth and cell division activity, while the cationic surfactant exhibits much lower toxicity levels. The research employed a technique called random amplification of polymorphic DNA as a biomarker to assess genetic damage, highlighting the importance of understanding the environmental impact of the surfactants [117].

The findings suggest that the cationic surfactant could be a safer and more environmentally friendly alternative to sodium dodecyl sulfate, emphasizing the need for further research on the long-term effects of surfactants on plant health and ecosystems. Overall, the study underscores the importance of developing less harmful chemical products to protect both environmental and human health.

Amino acid-derived surfactants may be promising compounds for biotechnological applications in which antimicrobial activity is required and reducing mammalian cell toxicity is essential. Thus, the use of highly active compounds minimizes surfactants exposure to human cells and increases the safety of these systems [68].

### Novel approaches in the use of amino acid-based surfactants

#### Cationic vesicles

Cationic vesicles have been prepared using glycerolipid arginine-based surfactants. Those structures were able to encapsulate different drugs such as ciprofloxacin and 5-fluorouracil. This innovation is particularly significant in the pharmaceutical field, as it influences both the inherent properties of ciprofloxacin and the innate antibacterial characteristics of certain surfactants, such as diacylglycerol-arginine-based compounds [118]. Their use has shown promising results against various bacterial strains, including *E. coli*, *K. pneumoniae*, and *S. aureus* [118]. These dual pharmacological functions not only enhance the delivery of ciprofloxacin but also offer a novel approach over bacterial infections by combining the drug's efficacy with the antibacterial properties of the vesicle components [39,118].

Pérez et al. (2020) formulated cationic vesicles incorporating the cationic histidine-based surfactant DMHysNHC14 (Table 1, entry 20) and the anionic surfactant (C12C3Lys) and evaluated their antimicrobial potential [20]. They found that the vesicles with the higher proportion of cationic histidine-derived surfactant exhibited enhanced antibacterial activity against Gram-positive strains [20].

The balance between antimicrobial activity and biocompatibility is crucial to prevent cellular damage. Strategically modulating the cationic/anionic surfactant ratio in catanionic vesicles can significantly enhance the therapeutic potential of these aggregates by optimizing both antimicrobial efficacy and hemolytic activity [30,44]. This approach offers a promising way to reduce cytotoxicity while boosting antimicrobial activity, making it advantageous for future biomedical applications [93].

#### Synergistic formulation

Another novel approach to promote their applicability is to mix the surfactants with functional adjuvants. For instance, a new synergistic formulation consisting of hyaluronic acid together with a biocompatible cationic surfactant derived from lysine, impaired the microbial growth of *S. aureus, E. coli, P. aeruginosa, P. mirabilis*, and *C. albicans* by 85% [119]. This approach can be readily expanded to create new coatings for various silicone-based materials, thereby enhancing the use of biomaterials, medical devices, diagnostic biosensors, among other applications [119–120].

Particularly noteworthy is the multifunctional surfactant PAβN (Phe-Arg β-naphthylamide), which exhibits dual mechanisms of action: as a standalone agent it shows a MIC of 16 μg/mL against *P. aeruginosa*, while in combination with ciprofloxacin it enhances antibiotic potency eightfold (reducing ciprofloxacin's MIC from 1 μg/mL to 0.125 μg/mL) through simultaneous membrane permeabilization and efflux pump inhibition [121].

Shen et al. (2023) developed long-lasting antimicrobial agents through hierarchical assemblies of gallic acid with LAM (Table 1, entry 1). Their experiments demonstrated, in vitro and in vivo, that these compounds were highly selective for penicillin-resistant *E. coli* and *S. aureus*, as well as *C. albicans*, without inducing toxicity [122].

#### Nanoparticles

Antimicrobial effectiveness also depends on the formulation and delivery strategies. For instance, when encapsulated in zein nanoparticles, phenylalanine surfactants (e.g., PNHC12) lose effectiveness due to the strong molecular interactions with the carrier matrix (most likely by the aromatic coupling), whereas surfactants combining both cationic (Arg and Phe) and hydrophobic amino acids (e.g., PANHC12, C12PAM) were found partially effective through the balanced molecular properties [82–83].

Current research demonstrates that zein nanoparticle encapsulation reduces the antimicrobial potency of phenylalanine surfactants (PNHC12), with MIC values against *S. aureus* increasing fourfold from 8 μg/mL (free form) to 32 μg/mL (encapsulated), attributable to aromatic π-stacking interactions that limit bioactivity. In contrast, structurally optimized arginine-phenylalanine hybrid surfactants (C12PAM) maintain their antimicrobial efficacy post-encapsulation, demonstrating consistent MIC values of 4–8 μg/mL against both Gram-positive and Gram-negative pathogens (Table 1) [123].

These quantitative findings reveal three critical formulation principles: (1) Conventional encapsulation approaches may significantly impair antimicrobial activity (4–8 times MIC increases), (2) Molecular hybridization strategies can preserve bioactivity in delivery systems, and (3) Multifunctional surfactants enable remarkable antibiotic synergy (8–16 times efficacy enhancement). The data underscore the necessity for precision molecular design to balance formulation stability with antimicrobial performance, particularly for overcoming biological barriers in drug-resistant infections.

Recent advances in the use of cationic surfactants for various applications are necessary to take advantage from their extraordinary antimicrobial responses [124]. For instance, Pérez et al. (2023) loaded the surfactants in zein nanoparticles [123,125]. Arginine-phenylalanine-derived surfactants loaded in these carriers were found to maintain the cationic properties, while selectively disrupted the microbial membranes, preserving the eukaryotic cells [82–83].

The antimicrobial activity is accompanied by improved biocompatibility, as the nanoparticles have shown enhanced selectivity towards representative cell lines of skin and connective tissues [126–127]. Consequently, those nanoparticles are promising for applications in tissue repair, particularly in wound treatments, where their antimicrobial efficacy can be useful to fight against the microbial biofilms as well as promote the healing, minimizing damage to host tissues [83].

#### Niosomes

The incorporation of surfactants in niosomes highlights the broader potential of vesicular systems in addressing complex challenges in drug delivery [128]. These structures, owing to their stability and small size, have shown significant antimicrobial potential in pharmaceutical applications [129–130]. This was demonstrated in a study involving the development of antimicrobial niosomes, using lidocaine and phenylalanine derivatives as primary components [128].

Phenylalanine-based surfactants formulated in niosomes enhanced the bactericidal effect against Gram-positive bacteria due to the affinity between the membrane of the microorganism and the formulation [131–132]. By combining the antimicrobial properties of these surfactants with the anesthetic effects of anesthetics such as lidocaine, these structures become a novel alternative to tissue repair and infection management. Their dual functionality positions them as promising candidates for future biomedical applications, particularly in wound healing and antimicrobial therapies for resistant species [128].

In addition to the pharmaceutical industry being constantly challenged in the discovery for new drugs, the food industry also faces constant challenges in the search for antimicrobial preservatives and food safety [124]. In this context, an example of an innovative, effective solution using amino acid-derived surfactants is the antimicrobial packaging [133]. A film containing only silver (specifically IONPURE IPL) has been compared to others combining silver and LAE. The material containing LAE caused a reduction in the growth of *S. enterica* and *P. putida*, while this effect was not observed in the material containing only silver. Therefore, LAE reinforced the package bioactivity [134].

These results are of great relevance given the growing demand for biocompatible, biodegradable, and antimicrobial agents effective against opportunistic pathogens [10,135]. The need for innovative solutions is essential, as conventional treatments are showing to be gradually ineffective to combat the resistant strains [136–138].

## Conclusion

Amino acid-derived surfactants demonstrate significant potential as next-generation antimicrobial agents, combining potent activity with low resistance development. Their unique structural properties, particularly cationic arginine-based compounds (e.g., LAE, LAM), enable effective membrane disruption, with MIC values ranging from 8 to 64 µg/mL against Gram-negative pathogens through strong electrostatic interactions with microbial membranes.

The incorporation of aromatic amino acids like tryptophan further enhances biofilm penetration, with studies showing a 40% deeper membrane insertion compared to conventional surfactants. Critically, the amino acid-derived surfactants exhibit a low propensity for resistance development, requiring 20–30 bacterial generations to achieve only modest (2–4 times) MIC increases, making them particularly valuable in the face of rising antimicrobial resistance.

In addition to their direct antimicrobial effects, amino acid-derived surfactants show remarkable synergy with existing antibiotics, improving ciprofloxacin efficacy by 8-fold against resistant strains. Their biocompatibility (HC_50_ > 200 µg/mL) and biodegradability further support their use in clinical and industrial applications, from topical formulations to nanoparticle-based drug delivery systems.

Future research should prioritize in vivo validation and the development of smart delivery systems, such as pH-responsive or biofilm-targeting formulations, to fully realize their therapeutic potential. With their multifunctional capabilities and sustainable profile, amino acid-derived surfactants represent a compelling alternative to traditional antimicrobials in our ongoing battle against drug-resistant infections.

## Data Availability

Data sharing is not applicable, as no new data were generated or developed in this study.
